# Possible link between *Toxoplasma gondii* and neurodegenerative diseases

**DOI:** 10.4314/ahs.v25i1.6

**Published:** 2025-03

**Authors:** Rugıyya Samadzade, Salih Maçin, Ahmed Moustapha Nsangou, Haluk Gumus, Neriman Akdam, Figen Guney, Sevil Kurban, Serefnur Ozturk, Duygu Findik

**Affiliations:** 1 Selcuk University Faculty of Medicine, Department of Medical Microbiology, Konya, Turkey; 2 Selcuk University Faculty of Medicine, Department of Neurology, Konya, Turkey; 3 Selcuk University Faculty of Medicine, Department of Biostatistics, Konya, Turkey; 4 Necmettin Erbakan University Medical Faculty, Department of Neurology, Konya, Turkey; 5 Necmettin Erbakan University Medical Faculty, Department of Medical Biochemistry, Konya, Turkey

**Keywords:** Alzheimer's disease, Electrochemiluminescence, Multiple Sclerosis, Parkinson's disease, *Toxoplasma gondii*

## Abstract

**Background:**

*Toxoplasma gondii* is one of the most common causes of zoonotic parasitic diseases in the world. Neurodegenerative diseases (Parkinson's, Alzheimer's, Multiple Sclerosis) are relatively common and create significant social and health problems. As a result of the studies, it has been hypothesized that the neuro-inflammation caused by *Toxoplasma gondii* may be responsible for the development of various neurodegenerative diseases.

**Objective:**

Serum samples of 300 patients with neurodegenerative diseases and 100 healthy volunteers were evaluated for the presence of *Toxoplasma gondii* IgG and IgM antibodies by Electrochemiluminescence method. A sociodemographic questionnaire was applied to the patients. Results were evaluated statistically.

**Results:**

*Toxoplasma gondii* IgG positivity was detected in 172 (57.3%) of 300 serum samples taken from the patient groups. Seropositivity was found in 42 (24.4%) patients with multiple sclerosis, 60 (34.9%) patients with Parkinson's disease and 70 (40.7%) patients with Alzheimer's disease. In the control group IgG was positive in 36 (36 %) subjects but IgM antibody positivity was not detected.

**Conclusions:**

According to the study results, there was a statistically significant relationship between the presence of *Toxoplasma gondii* seropositivity and Parkinson's and Alzheimer's diseases, but there was no a statistically significant relationship in multiple sclerosis patients.

## Introduction

*Toxoplasma gondii* is an obligate intracellular protozoan that infects humans and warm-blooded animals. It is transmitted to humans by ingesting the parasite's oocysts, scattering around with feces of cats, or consuming meat and meat products from infected animals^1^. Depending on contact with cats, eating and hygiene habits, 90% of the population can be infected^2^.

The prevalence of the parasite was found to be high globally. According to the results of studies, the prevalence of *T.gondii* in Brazil and France is higher than in countries such as South Africa and Turkey. *T.gondii* positivity was found to be over 50% in many European countries^3^. According to Turkey Ministry of Health guidelines associated with zoonotic diseases; It has been stated that the rate of *T.gondii* seropositivity is 30-60 % according to the regions and it is around 40% on average^4^.

Neurodegenerative diseases are a group of diseases that cause loss of nervous system functions due to progressive loss of neurons. In addition, they are characterized by filamentous structures within neuronal cells as well as neuronal loss^5^. The prevalence of Alzheimer's and Parkinson's diseases under the age of 40 indicates that aging is an important risk factor for these diseases^6^,^7^. Multiple sclerosis (MS), on the other hand, is a neurodegenerative disease that occurs at an early age and is relatively common, creating significant social and health problems. The treatment of these diseases is limited. Symptomatic treatment of MS and Parkinson's diseases is relatively successful. However, available treatments are extremely limited in terms of effectiveness in Alzheimer's patients^8^,^9^.

*T.gondii*, which is a powerful stimulator of the immune system, affects immune system mediators in the brain; It can also directly cause the production of cytokines, microglia cells, astrocytes, CD4 + and CD8 + cells. It is reported that neuronal degeneration caused by cytokine-related neuroinflammation also plays a key role in the pathogenesis of chronic neurodegenerative diseases. It has been stated that it causes a more intense cytokine-related inflammatory response in host cells depending on the form of the parasite (tachyzoites, bradyzoites)^10^.

In animal studies, it has been observed that oral or peritoneal administration of *T.gondii* genotypes I and III causes atrophy or hypoplasia in some parts of the gastrointestinal tract and death (hypertrophy) of some myenteric neurons. Therefore, it is thought that all these abnormalities in the organs associated with chronic *T.gondii* infection lead to the initiation of similar neuroinflammatory processes in the brain, which leads to increasing damage^11^.

Although *T.gondii* is a zoonotic infection showing widespread in Turkey, the knowledge about seroprevalence of *T. gondii* in neurodegenerative diseases is limited. For this reason, it was aimed to determine the seroprevalence of *T.gondii*. In addition, according to the results obtained from the study, it was aimed to evaluate the possible relationship between Parkinson's, Alzheimer's and multiple sclerosis and *T.gondii* together with sociodemographic characteristics.

## Subjects and methods

### Research group and method

Totally 300 patients who applied to Selcuk University Faculty of Medicine Neurology outpatient clinic and Necmettin Erbakan University Medical Faculty Neurology outpatient clinic between June 2018 and June 2019 and diagnosed with Parkinson's, Alzheimer's and Multiple sclerosis diseases and also 100 healthy volunteers without a history of neurodegenerative disease were included in the research.

A questionnaire form including cat contact, raw / undercooked meat consumption, hygiene (hygiene) habits and other sociodemographic characteristics was applied to both the patients and the control group. From the participants in the survey; “Have you been diagnosed with *Toxoplasma gondii* (IgM or IgG) positivity before?”, “Have you kept a cat at home?”, “How do you consume meat products?”, “What are your personal hygiene habits?”, “Where do you live? “What is the infrastructure of the place like?” and other questions were asked to be answered. The patient group's cat feeding, hygiene habits and meat consumption patterns in the period before the onset of neurodegenerative diseases were questioned.

*T. gondii* IgM and *T. gondii* IgG kits based on Electrochemiluminescence (ECL) method were used for diagnosis (Roche cobas e611, Sweden). Roche cobas e 601 *Toxo IgM/Toxo IgG* Reagent kits (M, R1, R2) are placed in the reagent disk of the analyzer. Foam formation was avoided when placing the reagents. The system automatically regulated the temperature of the reagents and the opening and closing of the bottles. All reagents were checked and the sensor cover of the analyzer was closed. Calibrators are placed in the sample area. All information required to calibrate the test was automatically read into the analyzer. Finally, the samples were introduced and loaded into the Roche cobas e 601 analyzer. The analyzer automatically calculated the analyte concentration of each sample in IU/mL.

The results of the samples were given in the form of threshold index (signal sample / threshold) as well as reactive (positive) or non-reactive (negative).

Samples with a threshold index <0.8 COI were considered non-reactive (negative) for the *T. gondii* IgM test. Samples with a threshold index of ≥1.0 COI were considered reactive (positive) for the *T. gondii* IgM test. Samples with concentrations <1 IU/mL were evaluated as non-reactive (negative) in the IgG test against Elecsys *T.gondii*. Samples with concentrations ≥ 3 IU/mL were considered reactive (positive) for IgG antibodies against T.gondii.

### Statistical Analysis of Data

The results obtained in the study were analyzed with the SPSS 21.0 package program. For each group, the Chi-Square Test and Fisher Excat test were applied to test whether there is relationship between *T.gondii* and cat feeding, consumption of meat products and hygiene. Binary logistic regression analysis was used to determine whether age, gender and the presence of *T.gondii* seropositivity variables are a risk factor in diseased individuals and odds ratio (OR, 95% OR Confidence Interval) values were calculated The analyzes is evaluated at the 5% significance level (95% confidence level). p< 0.05 is accepted statistically significant.

## Results

300 patients and 100 control samples were included in the study. 146 (48.7%) of the 300 patients were male, 154 (51.3%) were female. In the control group, 33 (33%) men and 67 (67%) women were included in the study. When the patient and control groups are evaluated as a whole; in the control group, the minimum age is 21, the maximum age is 88, in Multiple Sclerosis patients, the minimum age is 16 and the maximum age is 69, in Parkinson's patients, the minimum age is 19 and the maximum age is 94, in Alzheimer's patients is 55 and the maximum age is 90.

*T.gondii* is considered to be positive if at least one of *T.gondii* IgM and *T.gondii* IgG antibodies is positive. T.gondii IgM and/or *T.gondii* IgG were positive in 214 (71.3 %) of a total of 300 patient serum. *T.gondii* IgG positivity was detected a total of in 208 (52%) of patient and control group samples. *T.gondii* IgG was found to be positive in 172 (57.3%) patients, 42 (24.4%) with multiple sclerosis, 60 (34.9%) with Parkinson's disease and 70 (40.7%) with Alzheimer's disease. In addition, Toxoplasma IgG positivity was detected in 36 (36 %) of the control group. Presence of anti *T.gondii* IgM antibodies were found to be positive in 6 (2%) of the patient's serum. Anti-*T.gondii* IgM antibody positivity was not detected in the the control group. In 4 (1.3%) patients, both IgM and IgG positivity were detected.

In Table 3, p- values for the chi-square test are given. According to chi-square test, relationship between the presence of *T.gondii* seropositivity and Parkinson's disease was found to be statistically significant at p<0.0001 and α= 0.05. In addition, relationship between the presence of *T.gondii* seropositivity and Alzheimer's disease was found to be statistically significant with α = 0.05 and p < 0.0001. However, relationship between the presence of *T.gondii* and seropositivity in multiple sclerosis disease was not found to be statistically significant (p = 0.248).

**Table 3:** *T.gondii* (IgG and IgM) seropositivity in neurodegenerative diseases (Parkinson, Alzheimer, Multiple Sclerosis)

Parkinson Diseases			Parkinson	Control	Total	p-value
*Toxoplasma gondii*	Positive	N	62	36	98	p<0.0001
		%	63.3	36.7	100	
	Negative	N	38	64	102	
		%	37.3	62.7	100	
	Total	N	100	100	200	
Alzheimer Diseases			Alzheimer	Control	Total	p-value
*Toxoplasma gondii*	Positive	N	72	36	108	p<0.0001
		%	66.7	33.3	100	
	Negative	N	28	64	92	
		%	30.4	69.6	100	
	Total	N	100	100	200	
Multiple Sclerosis Diseases			Multiple Sclerosis	Control	Total	p-value
*Toxoplasma gondii*	Positive	N	44	36	80	0.248
		%	55	45	100	
	Negative	N	6	64	120	
		%	46.7	53.3	100	
	Total	N	100	100	200	

Age, gender and the presence of *T.gondii* seropositivity, were determined to be risk factors in individuals with Parkinson's disease (p<*α*= 0.05). Male gender increased the risk of Parkinson's disease by 4.28 times (p<0.0001). Parkinson's disease was 1.05 times higher when the age variable increases by 1 year (p<0.0001). Also, the presence of *T.gondii* seropositivity increased the risk of Parkinson's disease by 2.48 times (p=0.008< α= 0.05).

According to the chi-square analysis in Table 6, relationship between *T.gondii* seropositivity (Alzheimer diseas) and cat contact was statistically significant with p=0.010 at α= 0.05 significant level. However, relationship between *T.gondii* seropositivity (Parkinson's and multiple sclerosis diseas) and cat contact was not found to be statistically significant (p=0.421 and p=0.078).

**Table 6:** *T.gondii* seropositivity in neurodegenerative diseases (Parkinson, Alzheimer, Multiple Sclerosis) and its distribution by contact with cats

Cat contact			Parkinson			Alzheimer			Multiple Sclerosis		
			Yes	No	Total	Yes	No	Total	Yes	No	Total
***Toxoplasma gondii***	**Positive**	**N**	18	20	38	19	9	28	16	40	56
		**%**	47.4	52.6	100	67.9	32.1	100	28.6	71.4	100
	**Negative**	**n**	23	39	44	26	45	71	21	23	44
		**%**	37.1	62.9	100	36.6	63.4	100	47.7	52.3	100
	**Total**	**n**	41	59	100	45	54	99	37	63	99
**p-value**			0.421			0.010*			0.078		

* p<α= 0.05 relationship between variables statistically significant

According to the chi-square analysis in Table 7, the relationship between *T.gondii* seropositivity (Parkinson's, Alzheimer's and multiple sclerosis disease) and meat consumption status (under or overcooked) was not found to be statistically significant (p=0.615, p=0.120 and p=0.173).

**Table 7:** *T.gondii* seropositivity in neurodegenerative diseases (Parkinson's, Alzheimer's, Multiple Sclerosis) and its distribution by consumption of less or more cooked meat

Meat consumption status			Parkinson			Alzheimer			Multiple Sclerosis		
			Under cooked	Over cooked	Total	Under cooked	Over cooked	Total	Under cooked	Over cooked	Total
*Toxoplasma gondii*	Positive	n	14	23	37	16	12	28	23	33	56
		%	37.8	62.2	100	57.1	42.9	100	41.1	59.9	100
	Negative	n	28	34	62	27	45	72	25	19	44
		%	45.2	54.8	100	37.5	62.5	100	56.8	43.2	100
	Total	n	42	57	100	43	57	100	48	52	100
p-value			*0.615*			*0.120*			*0.173*		

* p<α= 0.05 relationship between variables statistically significant

According to the chi-square analysis in Table 8, the relationship between *T.gondii* seropositivity (Parkinson's and Alzheimer's disease) and hygiene habits was not found to be statistically significant (p=0.999 and p=0.219). Since only Good-Moderate was answered to the question of hygiene habits for Multiple Sclerosis disease, analysis was not performed.

**Table 8:** *T.gondii* seropositivity in neurodegenerative diseases (Parkinson, Alzheimer, Multiple Sclerosis) and distribution aocording to hygiene habits

Hygiene			Parkinson			Alzheimer			Multiple Sclerosis		
			Good-Moderate	Poor	Total	Good-Moderate	Poor	Total	Good-Moderate	Poor	Total
*Toxoplasma gondii*	Positive	N	36	2	38	24	3	27	56	0	56
		%	94.7	5.3	100	88.9	11.1	100	100	0.0	100
	Negative	N	59	3	62	54	18	72	44	0	44
		%	95.2	4.8	100	75	25	100	100	0.0	100
	Total	N	95	5	100	78	21	99	100	0	100
p-value			*0.999*			*0.219*			-		

* *p<α = 0.05* relationship between veriables statistically significant

According to the chi-square analysis in Table 9, relationship between *T.gondii* seropositivity of the control group and cat contact, meat consumption status (under or overcooked) and hygiene habits was not found to be statistically significant (p=0.154, p=0.309 and p>0.999).

**Table 9:** *T.gondii* seropositivity in the control group and distribution according to cat feeding, consumption of meat products and hygiene habits

			Control			Control			Control		
			Cat contact		Total	Meat consumption status		Total	Hygiene		Total
			Yes	No		Under cooked	Over cooked		Good-Moderate	Poor	
*Toxoplasma gondii*	Positive	n	23	41	64	14	50	64	63	1	64
		**%**	35.9	64.1	100	21.9	78.1	100	98.4	1.6	100
	Negative	n	19	17	36	12	24	36	35	1	36
		%	52.8	47.2	100	33.3	66.7	100	92.7	2.8	100
	Total	n	42	58	100	26	74	100	98	2	100
p-value			*0.154*			*0.309*			*p>0.999*		

* p<α= 0.05 relationship between variables statistically significant

## Discussion

The tachyzoite forms of *T.gondii* invade neurons, astrocytes, brain cells containing microglial cells, and Purkinje cells in the cerebellum. Intracellular tachyzoites manipulate the signaling pathways of transduction mechanisms involved in immune cell maturation and antimicrobial effector functions^12^. Torgerson et al. research supported the idea that cysts of *T.gondii* affect not only the neurons where they are localized, but also the host cell cytoplasm and some axons, thereby interfering with neuronal functions^13^.

Two genes encoding tyrosine hydroxylase, which is the restriction enzyme in dopamine synthesis, were found in the brain. The parasite may cause degeneration of dopamine-producing neurons in the brain as a result of high expression of these enzymes in the chronic phase (bradyzoite form)^14^,^15^,^16^. This neurodegenerative mechanism formed by the degeneration of these neurons that synthesize dopamine by *T. gondii* and the contribution of proinflammatory cytokines may cause neurodegenerative diseases^17^,^18^.

*Toxoplasma gondii* IgG antibodies in the Parkison's diseases and control groups were found to be 42.3% in patients and 22.5% in the control group respectively, and were statistically significant (p = 0.006)^19^. Fallahi et al. according to the results of the PCR test, found a statistically significant difference between toxoplasma infection (19.3%) and (10.4%) control groups (p = 0.002). According to their research results, they concluded that toxoplasma infection is not only a risk factor for Parkinson's disease^20^.

In our study, relationship between the *T.gondii* seropositivity and Parkinson disease is statistically significant. In addition, relationship no significant was found between *T.gondii* seropositivity and cat contact and meat consumption status (under or overcooked) and hygiene habits in Parkinson's disease.

As a common result in studies conducted to investigate the relationship between Parkinson's disease and *T.gondii;* dopamine produced by *T.gondii* is thought to cause degeneration and neurogenic inflammation of dopamine-producing neurons, resulting in Parkinson's disease. In addition, more comprehensive studies should be conducted considering that the positive correlation between toxoplasmosis and Parkinson's disease may lead to new approaches for the treatment of Parkinson's patients.

According to epidemiological studies, some risk factors for Alzheimer's etiology have been identified^21^. The common form of Alzheimer's disease (sporadic form) is a multifactorial disease that may include bacterial and viral pathogens^22^. Many infectious pathogens have been isolated from the brain tissue of Alzheimer's patients^23^. Pearce et al., suggested that some inflammatory cytokines such as IFN-qamma released after *T.gondii* infection cause degeneration of dopamineproducing neurons, causing Alzheimer's^24^.

In a study conducted on 34 patients with Alzheimer's disease and 37 healthy volunteers, 44.1% of and 24.3% of controls were found to be positive for anti-*T.gondii* IgG. According to the results, a statistically significant relationship was found between IgG positive titer and Alzheimer's disease^25^.

Increased microglial cells, cytokines, reactive oxygen species, complement factors, neurotoxic secretory products and free radicals when exposed to inflammatory stimulants, describes that it increases inflammatory responses by releasing various mediators^26^,^27^,^28^,^29^. Moreover, many of these mediators have been reported to stimulate amyloid precursor protein (APP) accumulation and contribute to neuronal death in AD. Furthermore, the results of this study showed that it can create a vicious cycle for the stimulation of microglial cell activation, inflammatory mediator release and APP production^30^.

In our study, relationship between the presence of *T.gondii* seropositivity and Alzheimer's disease was found to be statistically significant. In addition, relationshipa significant was found between *T.gondii* seropositivity and cat feeding in Alzheimer's patients. However, relationship no significant was found between the *T.gondii* seropositivity of Alzheimer's patients and their meat consumption (under or over cooked) and hygiene habits.

Multiple sclerosis is a chronic and multifocal demyelinating disease, a central nervous system disease that mainly affects young adults. Autoimmune diseases such as MS are thought to result from a combination of genetic susceptibility and environmental factors^31^. It is thought that various infectious agents such as human cytomegalovirus (CMV), Epstein-Barr virus (EBV), Human Herpes Virus 6 and, *Chlamydia pneumoniae* and potential environmental or causal factors play a role in the pathogenesis of MS^32^,^33^.

Parasite infections may be associated, with the pathogenesis of autoimmune diseases such as Multiple sclerosis. This phenomenon was first proposed by Strachan in 1989 with the “hygiene hypothesis”. With this hypothesis, it has been suggested that repeated exposure to infectious agents in childhood may protect against the development of allergies and autoimmunity^34^.

In the study conducted Correala et al. it was reported that IL-10 has a positive feedback effect on the production of B cells that reduce autoimmune reactions by making a regulatory function in Multiple sclerosis patients. These studies largely support the hygiene hypothesis, which suggests that parasite infections may have protective effects against autoimmune diseases^35^. It was found that in infected mice the parasite not only triggered Interferon (IFN)-production in T helper cells, but also IL-10 demonstrated regulatory activities in the acute and chronic phase of infection^36^,^37^. To date, there is no data available on any studies with experimental animals to understand the role of *T. gondii* in the pathogenesis of MS. In addition, there is no scientific data regarding possible humoral and cellular differences between *T. gondii*-infected and non-infected patients.

In our study, relationship between the presence of *T.gondii* seropositivity and seropositivity in multiple sclerosis disease was not found to be statistically significant. In addition, relationshipno significant was found between *T.gondii* seropositivity and cat contact, meat consumption status (undercooked or overcooked), and hygiene habits in multiple sclerosis patients.

## Conclusion

This study is one of the first comprehensive studies investigating the possible relationship between *T. gondii* and neurodegenerative diseases in Turkey. Further studies are needed in the future to validate the results of our study and to understand the underlying pathophysiological mechanisms. This will provide insight into new mechanisms underlying the pathogenesis of neurodegenerative diseases and contribute to the development of potential immune-modulating therapies for neurodegenerative diseases and other autoimmune diseases.

## Figures and Tables

**Table 1:** Gender Distribution of patient and control group

Age	Male				Female			
	n	Min.	Max.	Mean.±Std. Dev.	n	Min.	Max.	Mean.±Std. Dev.
Control	33	21,0	81,0	53,4±18,5	67	21,0	88,0	50,7±18,3
Multiple Sclerosis	33	16,0	62,0	38,5±12,3	67	18,0	69,0	38,6±10,6
Parkinson	67	31,0	31,0	65,5±12,6	33	19,0	94,0	64,4±13,2
Alzheimer	46	55,0	55,0	73,4±8,9	54	59,0	90,0	74,9±8,1

**Table 2:** Distribution by age in patient and control groups

Age	N	Min.	Max.	Med.±Std. Dev.
Control	100	21,0	88,0	51,6±18,3
Multiple Sclerosis	100	16,0	69,0	38,6±11,1
Parkinson	100	19,0	94,0	65,2±12,8
Alzheimer	100	55,0	90,0	74,2±8,5

**Table 4:** Logistic regression analysis according to age, gender and the presence of *T.gondii* seropositivity for Parkinson Diseases

Parkinson Diseases	Odss Ratio (OR)	95% C.I. for OR	p-value
Age	1.05	1.02-1.07	*p<0.0001*
Gender	4.28	2.20-8.31	*p<0.0001*
The presence of *T.gondii* seropositivity	2.48	1.27-4.86	0.008*
Constant	0.020		*p<0.0001*

* *p< α= 0.05* OR statistically significant

**Table 5:** Logistic regression analysis according to age, gender and the presence of *T.gondi* seropositivity for Alzheimer Diseases

Alzheimer Diseases	Odss Ratio (OR)	95% C.I. for OR	p-value
Age	1.15	1.10-1.21	*p<0.0001*
Gender	1.99	0.894.46	*0.090*
The presence of *T.gondii* seropositivity	3.70	1.66-8.22	0.001*
Constant	0.0007		*p<0.0001*

* *p<α= 0.05* OR statistically significant

## Financing/Support

This study was supported by Selcuk University Coordinatorship of Research Projects (Project No: 18202030)
